# Effect of physical exercise on executive function in individuals with substance use disorder: A systematic review and meta-analysis

**DOI:** 10.1097/MD.0000000000049771

**Published:** 2026-07-17

**Authors:** Jiawei Chen, Xiaofei Zhang, AV Kabachkova, Siqin Zeng, Wenwu Xiao

**Affiliations:** a Department of Rehabilitation Medicine, Renhe Hospital Affiliated to China Three Gorges University, Yichang, China; b Faculty of Physical Education, National Research Tomsk State University, Tomsk, Russia; c Department of Rehabilitation Medicine, Three Gorges Hospital Affiliated to Chongqing University, Chongqing, China; d Department of Fundamental Psychology and Behavioral Medicine, Siberian State Medical University, Tomsk, Russia; e Hunan Traditional Chinese Medical College, Zhuzhou, China; f Brain Neuromodulation of Yichang Key Laboratory, Yichang, China.

**Keywords:** executive function, exercise, meta-analysis, substance use disorder, systematic review

## Abstract

**Background:**

The increasing number of individuals with substance use disorder (SUD) has a serious impact on individuals, families, and society. As a complementary therapy, physical exercise has been used in the rehabilitation of individuals with SUD. This study systematically aimed to evaluate the effect of physical exercise interventions on enhancing executive function in individuals with SUD.

**Methods:**

Electronic databases were searched, covering the period from their inception to December 26, 2024. The Cochrane Risk of Bias Assessment Tool was utilized to assess the methodological quality of the included literature. Data were extracted from study graphs using GetData Graph Digitizer 2.26, and Stata 17.0 was also employed to conduct analysis. Grading of recommendations assessment, development and evaluation pro was utilized to evaluate the quality of evidence of outcome indicators.

**Results:**

Nine studies, encompassing 752 participants, were included in the analysis. Compared with the control group, physical exercise improved inhibition (Hedges’s *g* = 0.47, 95% CI: 0.29 to 0.65, *P* < .001) and working memory (Hedges’s *g* = 0.42, 95% CI: 0.16 to 0.69, *P* = .002), but did not significantly affect cognitive flexibility (Hedges’s *g* = 0.51, 95% CI: −0.22 to 1.24, *P* = .170). Subgroup analysis revealed that physical exercise with single time of 46 to 60 minutes (Hedges’s *g* = 0.39, 95% CI: 0.05 to 0.73, *P* = .024), 5 times per week (Hedges’s *g* = 0.45, 95% CI: 0.07 to 0.83, *P* = .020), duration of 8 weeks (Hedges’s *g* = 0.46, 95% CI: 0.14 to 0.77, *P* = .004) may improve working memory in individuals with SUD (these subgroup findings are exploratory and should be interpreted as hypothesis-generating).

**Conclusion:**

Evidence from this study suggests physical exercise may enhance inhibition and working memory in individuals with SUD. However, the improvement in cognitive flexibility requires further investigation in future studies.

## 1. Introduction

Substance use disorder (SUD) includes various addiction-related mental disorders resulting from prolonged psychoactive substance consumption, posing a significant global public health challenge. The World Drug Report 2020 by the United Nations Office on Drugs and Crime reported 269 million drug users worldwide.^[1]^ The 2020 National Survey on Drug Use and Health estimated that approximately 4.03 million individuals were diagnosed with SUD based on the Diagnostic and Statistical Manual of Mental Disorders-5 criteria.^[2]^ The individuals with SUD often exhibit severe physiological issues and pathological behavioral patterns due to chronic substance use manifesting as changes in physiological function, such as altered brain circuitry.^[3]^

SUDs are chronic, relapsing conditions characterized by executive dysfunction.^[4]^ Numerous electrophysiological studies have shown that individuals with SUD often exhibit impaired executive function.^[5,6]^ Miyake et al.^[7]^ Proposed a three-factor model of executive function, which includes inhibition, working memory, and cognitive flexibility. Inhibition is a central executive function that involves resisting initial impulse and instead acting more wisely and intentionally. Working memory is essential for holding information and is critical for reasoning and problem-solving. Cognitive flexibility refers to the ability to adapt to changing demands, view situations from different perspectives, and switch tasks. These components are widely accepted as the core of executive function.^[8,9]^ The present study’s focus on executive function is based on Miyake’s three-factor model. These subcomponents are crucial for planning, decision-making, inhibitory control, and flexible information processing during complex tasks.^[10]^ Executive function has been suggested to play a crucial role in different aspects among individuals with SUD. Individuals with SUD may experience impaired executive function, which in turn hinders their ability to suppress drug cravings^[11]^ and relapse behavior,^[12]^ influences sound decision-making,^[13]^ and increases the likelihood of high-risk behavior. Conversely, improving executive function can help individuals with SUD manage stress and negative emotions more effectively.^[14]^ Enhanced executive function may also restore social function, bolster interpersonal relationships, and improve employment status, ultimately strengthening social resilience.^[15]^ These data suggest that executive function is critical for the treatment and rehabilitation of individuals with SUD, integral to preventing relapse, and essential for improving their quality of life and social functioning. Therefore, enhancing executive function through targeted interventions may offer new strategies for the treatment and management of SUD.

The evolving understanding of SUD has led to the investigation of various non-pharmacological interventions to enhance therapeutic outcomes. Among these strategies, physical exercise has garnered significant attention due to its cost, easy-to-use characteristics, and minimal side effects. Emerging evidence suggests that physical exercise can enhance executive function and serve as a safe and effective alternative to medication in mitigating cognitive decline.^[16,17]^ The potential mechanism involves physical exercise influencing the expression of neurotransmitters and neurotrophic factors,^[18]^ synaptic plasticity, and neurogenesis.^[19]^ Additionally, it modulates cognitive function by affecting inflammatory pathways^[20]^ and the cerebrovascular system.^[21]^

Although many studies have demonstrated that physical exercise positively influences executive function in individuals with SUD, the relationship between exercise “dose” and effectiveness remains ambiguous. In addition, whether physical exercise exerts a universal^[22]^ or selective^[23]^ impact on the subcomponents of executive function is contested. Some research indicates that exercise broadly enhances multiple cognitive domains, including executive function, in individuals with SUD. For instance, a randomized controlled trial (RCT) found that physical exercise significantly improved executive function in individuals with SUD.^[24]^ Similarly, another trial reported overall improvements in executive function among individuals with SUD following physical exercise.^[25]^ Conversely, several studies suggest that exercise selectively affects executive function subcomponents in individuals with SUD. For example, it has been suggested that physical exercise may not have a significant effect on working memory.^[26]^ Additionally, other studies have observed that physical exercise may negatively affect inhibitory functioning in individuals with SUD.^[27]^ Reviewing previous studies, the reason for these differing views may be due to the fact that the effects of physical exercise on executive function in individuals with SUD are influenced by a variety of factors, including intensity, frequency, duration, and single time of the physical exercise, as well as types of substance used. Therefore, further systematic analyses are necessary to elucidate the effects of physical exercise on executive function and its subcomponents in individuals with SUD.

This study is a meta-analysis on this topic to evaluate the impact of physical exercise interventions on executive function in individuals with SUD and to examine the effects and dosage relationships across different subcomponents of executive function. This study aims to explore the relationship between exercise dose parameters and executive function outcomes in individuals with SUD, providing preliminary evidence to inform future hypothesis-driven investigations of optimal exercise prescription.

## 2. Research methods

This study adhered to the requirements of the Preferred Reporting Items for Systematic Reviews and Meta-Analyses guidelines for systematic reviews and meta-analyses. The Preferred Reporting Items for Systematic Reviews and Meta-Analyses guidelines are a set of evidence-based recommendations for improving the quality of reporting in systematic reviews and meta-analyses. These guidelines offer a standardized framework for authors to prepare and report their systematic reviews, thereby enhancing transparency and reproducibility in research synthesis.^[28]^ The research protocol was registered on PROSPERO (No: CRD42024530109).

### 2.1. Population, intervention, comparison, outcomes, study design (PICOS) Strategy

This meta-analysis assessed the elements (e.g., frequency, duration, single exercise time, intensity) of exercise interventions based on subcomponent-specific outcome indicators of executive function. It explored the effects of exercise on various executive function subcomponents in individuals with SUD and investigated the potential dose-response relationship. The PICOS strategy is a systematic approach used to identify and select studies based on specific criteria related to the population, intervention, comparison, outcomes, and study design.^[29]^ It was employed to ensure a focused and methodical selection process for our meta-analysis, allowing us to target studies that are directly relevant to our research question and objectives. This strategy helps in minimizing bias and enhancing the rigor of our study selection. The PICOS strategy used in this study was detailed in Table 1.

**Table 1:** The PICOS strategy used in this study according to the components of population, intervention, comparison, outcomes, and study design.

Term	Description
Population	Individuals with SUD.
Intervention	Physical exercise interventions.
Comparison	Routine rehabilitation treatment or no physical exercise intervention.
Outcomes	Executive function-related outcomes, including inhibition, cognitive flexibility, and working memory.
Study design	Randomized controlled trials.

### 2.2. Literature search strategies

Data from the included literature were collated and statistically analyzed in accordance with the Guidelines for Writing Systematic Reviews.^[30]^ Searches were conducted in PubMed, EMBASE, the Cochrane Library, and the Web of Science (WoS) from each database’s inception to December 26 2024. The search strategy for the WoS database, as an example, is detailed below. Complete search strategies for all 4 databases (PubMed, EMBASE, Cochrane Library, and WoS) are provided in Supplementary File S1, Supplemental Digital Content 1. No language or regional restrictions were applied.

#1 TS = (SUD)

#2 (((AB = (substance abuse)) OR AB = (drug abuse)) OR AB = (drug addiction)) OR AB = (drug use disorder)

#3 #1 OR #2

#4 TS = (exercise)

#5 ((((((AB = (physical exercise)) OR AB = (physical activity)) OR AB = (training)

#6 #4 OR #5

#7 TS = (cognition)

#8 (((((AB = (executive function)) OR AB = (cognitive function)) OR AB = (inhibition)) OR AB = (inhibitory control)) OR AB = (switching function)) OR AB = (working memory)) OR AB = (cognitive flexibility)

#9 #7 OR #8

#10 TS = (randomized controlled trial)

#11 ((AB = (randomized)) OR AB = (controlled)) OR AB = (trial)

#12 #10 OR #11

#13 #3 AND #6 AND #9 AND #12

### 2.3. Literature inclusion criteria

Studies were included if they met the following criteria: randomized controlled trials; study subjects: participants diagnosed with SUD according to the International Classification of Diseases,^[31]^ the American Psychiatric Association statistical manual of mental disorders-5,^[32]^ or other validated clinical diagnostic criteria for SUD. And the target population including individuals with SUD in Mandatory Detoxification and Rehabilitation Center, Drug Rehabilitation Institute, residential treatment, outpatient programs, as well as mutual self-help groups such as Alcoholics Anonymous (AA) and Narcotics Anonymous (NA); interventions involving physical exercise in the treatment group, which may include aerobic, anaerobic, or mixed modalities, including any form of physical exercise (e.g., treadmill, cycling, running, bodyweight exercises, etc); control groups receiving routine rehabilitation treatment (in compulsory detoxification centers, it typically comprised structured daily schedules with health education, and supervised routines; in community-based programs, it involved less structured psychosocial support and voluntary health education sessions), no exercise intervention, no treatment, routine care, health education program, etc; and primary or partially primary outcome measures related to executive function, including inhibition, cognitive flexibility, and working memory.

### 2.4. Literature exclusion criteria

Studies were excluded if they met any of the following criteria: reviews, commentaries, animal research, nonintervention studies, conference papers, or duplicate publications; inconsistent intervention modalities or outcome indicators; incomplete experimental data that could not be calculated (extraction of the data revealed missing data on the outcome indicators measured before and after the intervention in the literature, and the original data were still not available after contacting the authors); co-existence of other severe physical and mental disorders including musculoskeletal disorders, major organic diseases, severe mental illness, cognitive impairment, psychiatric abnormalities or severe perceptual disorders; and studies lacking a control group were excluded.

### 2.5. Data extraction

Two researchers independently screened the literature, extracted the data, and cross-checked the results. Studies that required data extraction from figures using GetData Graph Digitizer 2.26 were documented; inter-rater agreement was assessed for a random subset (20%) of digitized values.^[33]^ Authors were contacted for raw data when figures were the only data source; studies for which data remained unavailable after author contact were excluded (see Section 2.4). Disagreements were resolved through consultation with a third researcher. Extracted data included basic literature information (e.g., authors, publication year, country), participant details (e.g., sample size, age, gender), exercise methods (e.g., frequency, duration, single exercise time, intensity, type) categorized based on previous research ^[34]^ and main outcome indicators. For studies employing multiple cognitive test instruments for a subcomponent of executive function, a predefined hierarchy was applied: the instrument with the highest frequency across included studies was selected for each subdomain (inhibition: Stop-Signal Test, Stroop Task; working memory: N-back Task, Digit Span; cognitive flexibility: Trail Making Test Part B, Wisconsin Card Sorting Test). Studies providing data only in graphical form were extracted using GetData Graph Digitizer 2.26; see above for validation procedures. Outcome indicator values were expressed as the mean and standard deviation (SD). Means and SD were extracted for each variable at baseline and at the end of the trial for both the intervention and control groups. For the one study^[35]^ that reported outcomes at 3 time points (1-month, 2-month, and 3-month), only the 3-month time point (end-of-intervention) was included in the primary analysis to avoid double-counting of participants and maintain independence of effect sizes (ES) across comparisons. When SDs within groups were not provided directly, the study utilized formulas from the Cochrane Handbook to calculate SDs from standard errors (SEs) of means (SEM) or 95% confidence intervals (CI) as follows:

SD = SEM × √n, where n is the sample size of the group; SD = √n × (CI_upper − CI_lower)/ (2 × *t*), where t is the critical value of the *t* distribution with n–1 degrees of freedom at the 0.025 level (for large samples, n ≥ 60, *t* ≈ 1.96).

### 2.6. Quality assessment

The risk of bias in the included literature was evaluated according to the Cochrane Handbook 5.1.0,^[36]^ considering factors such as random sequence generation, allocation concealment, blinding of participants and researchers, blinding of outcome evaluators, incomplete outcome data, selective reporting, and other biases. Each indicator was rated as “low risk of bias,” “uncertain risk of bias,” or “high risk of bias.” The quality of evidence for outcome indicators was assessed using the GRADEpro evidence grading system, which classifies evidence quality into 4 categories: “high,” “moderate,” “low,” and “very low.” Two investigators independently assigned quality ratings, with discrepancies resolved through consultation with a third investigator until consensus was reached.

### 2.7. Statistical analysis

Statistical analyses were performed using Stata 17.0 software (StataCorp, College Station). The metan package was used for meta-analytic pooling, and the metaninf command was used for leave-one-out sensitivity analyses. Effect sizes (ESs, i.e., Hedges’ *g*; with 95% CIs [95% CIs]) were calculated for the main outcomes based on pre-to-post-intervention change scores using the DerSimonian and Laird random effects model. For small numbers of studies (*k* < 5), the modified Hartung-Knapp-Sidik-Jonkman adjustment^[37]^ was applied, which imposes a lower bound of 1 on the scaling factor to obtain more conservative and better-calibrated CIs. Where feasible (*k* > 3), 95% prediction intervals were calculated to quantify the expected range of true effects in future studies.^[38]^ Heterogeneity was assessed with the I^2^ statistic and categorized as low (<25%), moderate (25%–75%), and high (>75%).^[39]^ Exercise dose variables were defined as follows: single exercise time (30–45 vs 46–60 minutes per session), exercise frequency (3 vs 5 sessions per week), and total exercise duration (4, 8, or 12 weeks). These cut-points were determined by the distribution of characteristics across included studies and represent descriptive categorizations rather than a priori hypothesis tests. All subgroup analyses were prespecified as exploratory and should be interpreted as hypothesis-generating. Galbraith plots were applied to visualize outliers with heterogeneity in the Meta-analysis. Sensitivity analysis was used to assess the stability of the combined results. When the number of studies was 10 or more, publication bias was assessed. Statistical significance was established at *P* < .05.

## 3. Results

### 3.1. Literature search results

**Figure 1.**
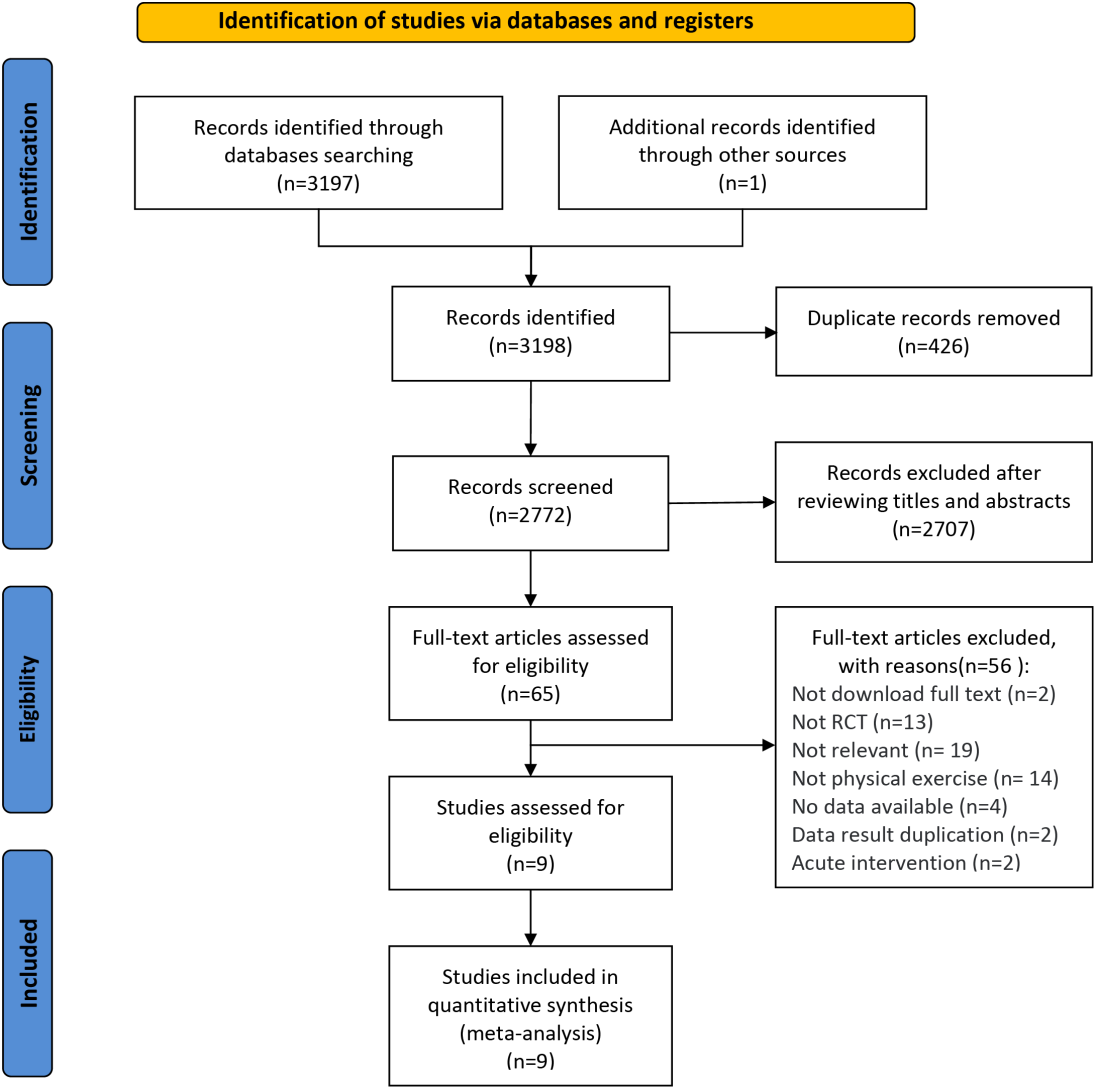
Flow chart of the literature screening process.

The literature search in PubMed, WoS, the Cochrane Library, and EMBASE databases initially retrieved 3197 relevant articles. One additional article was included after reference tracing. Subsequently, 426 duplicates were removed, and 2707 articles were excluded during the initial screening based on titles and abstracts. The remaining 65 articles underwent full-text screening, resulting in the exclusion of those not meeting the inclusion criteria. Ultimately, 9 studies were included in the meta-analysis, as illustrated in Figure 1.

### 3.2. Basic characteristics of the included literature

Nine studies were included. The studies comprised included 752 participants, primarily with alcohol or methamphetamine addiction. There was only one study that had a look at the individuals with alcohol use disorder. The exercise intensity in all studies was moderate. The exercise types in all studies were aerobic exercise. The exercise duration ranged from 4 to 12 weeks, with a frequency of 3 to 5 times per week, and a single exercise time lasting 30 to 60 minutes. (Table 2)

**Table 2:** Basic information of the literature included in the meta-analysis.

Study	Country	Yr	Type	Sample size (T/C)	Addictive substance	Diagnostic criteria	Duration of using substance	Genders(M/F)	Age T/C	Exercise frequency and duration (T)	Single exercise time(min)(T)	Exercise intensity (T)	Intervention type (T)	Intervention type(C)	Measurements
Dongshi Wang^[40]^	China	2017	RCT	25/25	Methamphetamine	DSM-IV	67.39 ± 43.49/85.13 ± 54.04 (mo)	44/6	32.20 ± 6.97/34.76 ± 7.96	3 times/week;12 weeks	30	Moderate	Cycling, jogging, or jump rope	Routine care	Inhibition (Go/No-go task); Working memory (N-back task)
Jingjing Liu^[25]^	China	2021	RCT	43/46	Methamphetamine	DSM-Ⅲ	(8.05 ± 3.84) /(7.82 ± 3.57) (yr)	0/89	29.43 ± 7.91/28.96 ± 8.27	5 times/week;12 weeks	40	Moderate	Including head movements, shoulder movements, chest movements, waist movements, upper limb movements, and lower limb movements	Routine treatment	Inhibition (Color-Word Stroop Test); Working memory (Memory for Persons Data)
Zhang Kai^[41]^	China	2018	RCT	34/32	Methamphetamine	DSM-V	78.69 ± 53.00(mo)	NA	33.29 ± 7.74	3 times/week;12 weeks	30	Moderate	Cycling, jogging, or jump rope	Routine treatment	Working memory (N-back task)
Ting Zhu^[35]^	China	2021	RCT	40/37	Methamphetamine	DSM-V	≥2 (yr)	NA	34.61 ± 5.12/34.94 ± 6.58	5 times/week;12 weeks	30-36	Moderate	Balance, chest expansion, kicking, horse-step stretching, bow step pushing, abdomen and back movements, and jumping	Compulsory drug withdrawal procedures without engaging in additional exercise	Inhibition (Go-no go task)
Xiao-xia Liu^[42]^	China	2021	RCT	142/146	Methamphetamine	DSM-V	7.32 ± 4.47/7.28 ± 3.39 (yr)	NA	31.27 ± 5.50/30.92 ± 5.00	5 times/week;8 weeks	60	Moderate	Treadmill	Health education	Cognitive flexibility (Switching task); inhibition (Stroop task); working memory (N-back task)
Xiao-xia Liu^[43]^	China	2021	RCT	23/23	Methamphetamine	DSM-V	8.08 ± 3.10/8.44 ± 3.62 (yr)	NA	28.80 ± 3.62/27.66 ± 3.66	5 times/week;8 weeks	60	Moderate	Running	Health education	Inhibition (Stroop task); working memory (N-back task)
Yuping Zhu^[44]^	China	2023	RCT	30/30	Methamphetamine	DSM-V	5.35 ± 2.32 (yr)	60/0	30.15 ± 3.24	5 times/week;8 weeks	40	Moderate	Cycling on a cycle ergometer, running on a treadmill, and using an elliptical machine	Routine treatment	Inhibition (Go/No-go task); working memory (Sternberg paradigm)
Pennington David L.^[45]^	USA	2022	RCT	15/15	Alcohol	DSM-V	NA	25/5	49.2 ± 11.0/52.8 ± 7.7	3 times/week;8 weeks	30	Moderate	Identical stationary recumbent bicycle	Gameplay control	Inhibition (D-KEFS Color-Word Interference Test Condition 3); cognitive flexibility (D-KEFS Trail Making Condition 4 (Letter-Number Sequencing) and Design Fluency Condition 3 (Switching Dots)); working memory (WAIS-IV Arithmetic and Digit Span)
Xiao-xia Liu^[46]^	China	2024	RCT	23/23	Methamphetamine	DSM-IV	8.08 ± 3.10/8.44 ± 3.62 (yr)	46/0	26.63 ± 3.27/27.91 ± 3.13	3 times/week;8 weeks	60	Moderate	Treadmill aerobics program	Health education	Inhibition (Stroop task); working memory (2-back) ; cognitive flexibility (Shift task)

C = Control group, DSM-Ⅲ = American Psychiatric Association’s Diagnostic and Statistical Manual of Mental Disorders, Third Edition, Fourth Edition (DSM-IV), Fifth Edition (DSM-Ⅴ), F = Female, HRmax = Maximum Heart Rate, HRR = Heart rate recovery, M = Male, NA = not applicable, T = Treatment group.

### 3.3. Assessment of methodological quality of the included literature

**Figure 2.**
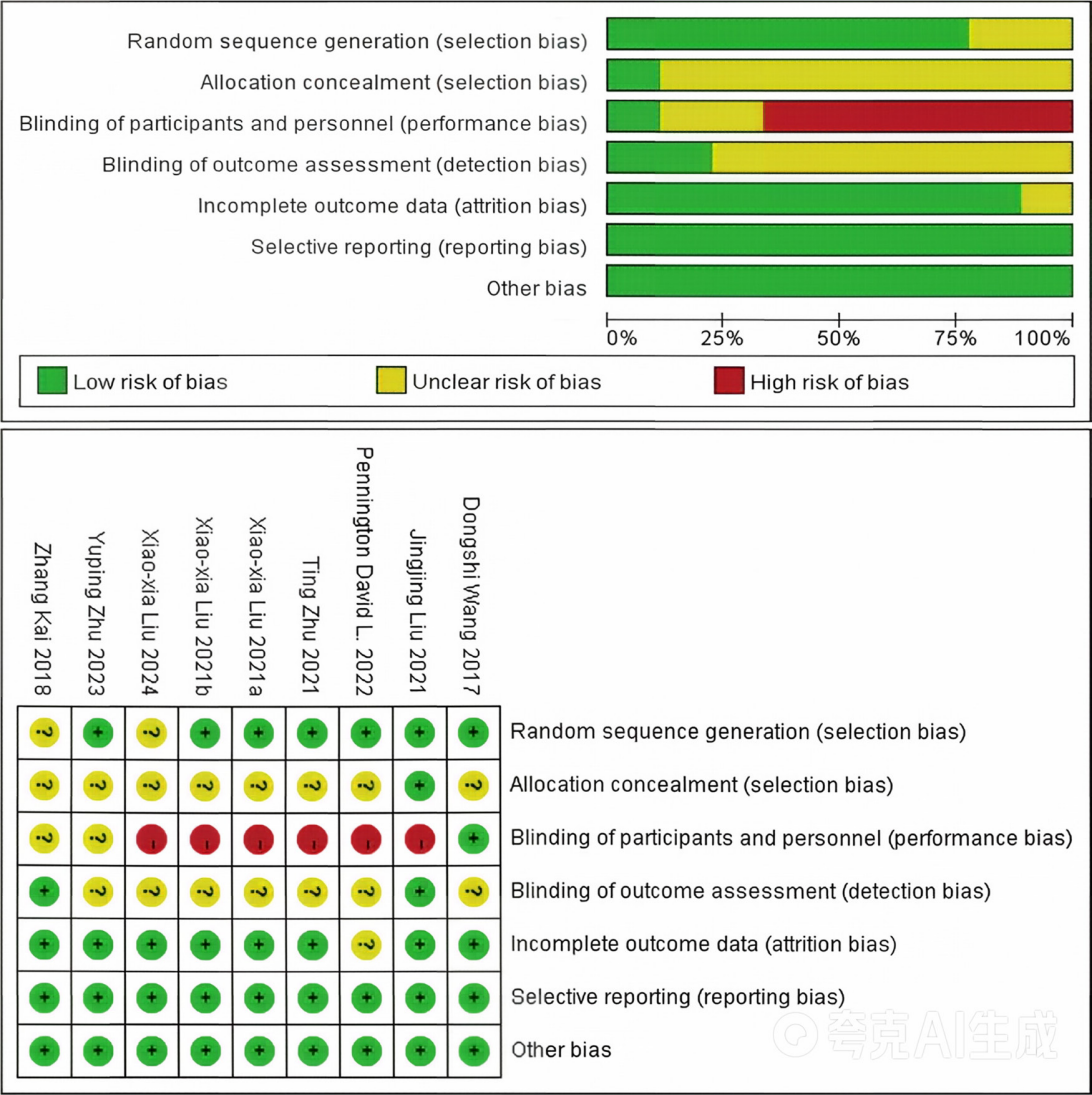
Risk of bias graph for the included studies.

Detailed randomization methods were described in 9 studies, with one mentioning allocation concealment, one reporting blinding of participants and investigators, two employing blinding of outcome evaluators, and the remainder having unclear or no blinding measures. All studies reported complete outcome data and reports, as illustrated in Figure 2.

### 3.4. Meta-analysis results

#### 3.4.1. Effects of physical exercise intervention on inhibition in individuals with SUD

**Figure 3.**
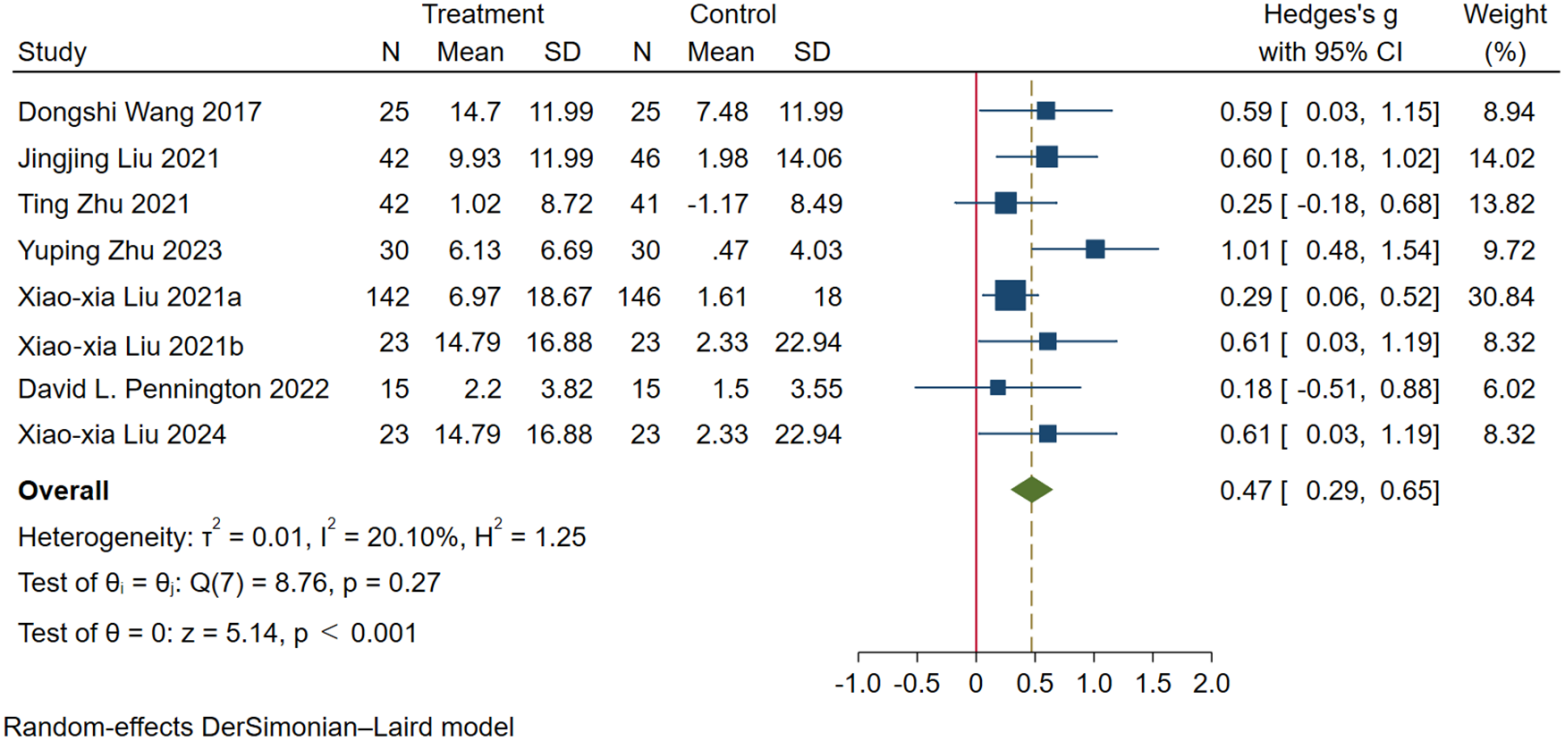
Forest plot of the effect of exercise on the inhibition. CI = confidence interval, SD = standard deviation.

Eight of the 9 included studies assessed the inhibition subcomponent of executive function ^[25,35,40,42–46]^, resulting in a total of 8 comparisons. The heterogeneity test indicated low heterogeneity among the included studies (*I*^2^ = 20.10%, *P* = .270). Compared with the control group, a statistically significant small-to-moderate effect was observed in the treatment group (Hedges’s *g* = 0.47, 95% CI: 0.29 to 0.65, *P* < .001). It suggested that physical exercise may improve inhibition (Fig. 3). The 95% prediction interval for the inhibition outcome ranged from 0.12 to 0.82, indicating that the true effect of physical exercise on inhibition in a future comparable study is expected to be positive.

A subgroup analysis of potential adjustment variables was conducted to identify possible sources of heterogeneity. Due to the absence of additional variables such as gender and duration of use in the extracted data, and with no significant age differences and only moderate intensity exercise reported in the relevant studies, the analysis focused on single exercise time, exercise frequency, and total exercise intervention duration. Exercise dose variables were defined as follows: single exercise time (30–45 vs 46–60 minutes per session), exercise frequency (3 vs 5 sessions per week), and total exercise duration (4, 8, or 12 weeks). These cut-points were determined by the distribution of characteristics across included studies and represent descriptive categorizations rather than a priori hypothesis tests. All subgroup analyses were prespecified as exploratory and should be interpreted as hypothesis-generating. As shown in Table 3, significant differences were observed between the treatment and control groups based on different single exercise times and exercise frequencies. However, differences were not statistically significant when the exercise duration was 4 weeks. With single exercise time lasting 30 to 60 minutes, 3 or 5 times per week, or exercise duration of 8 or twelve weeks, may improve inhibition in individuals with SUD (these subgroup findings are exploratory and should be interpreted as hypothesis-generating).

**Table 3:** Subgroup analysis of the effect of different exercise variables on the inhibition and working memory.

Subcomponents	Adjustment variables	Subgroup category	n/RCTs	Heterogeneity			The pooled effect	
				*Q*	P	*I^2^* (%)	Hedges’s g (95% CI)	P
Inhibition	Time	30–45 min	5	5.87	0.209	31.87	0.54 (0.26–0.81)	<0.001
		46–60 min	3	1.73	0.422	0.00	0.37 (0.17–0.57)	<0.001
	Frequency	3 times/wk	4	3.55	0.315	15.43	0.64 (0.33–0.96)	<0.001
		5 times/wk	4	2.51	0.473	0.00	0.37 (0.19–0.54)	<0.001
	Duration	4 wk	1	0.00	-	-	0.19 (-0.51 to 0.88)	0.604
		8 wk	4	6.66	0.084	54.93	0.58 (0.23–0.92)	0.001
		12 wk	3	1.55	0.461	0.00	0.47 (0.20–0.73)	0.001
Working memory	Time	30–45 min	4	8.93	0.030	66.39	0.43 (-0.02 to 0.87)	0.062
		46–60 min	3	3.55	0.169	43.73	0.39 (0.05–0.73)	0.024
	Frequency	3 times/wk	4	7.82	0.050	61.66	0.40 (-0.06 to 0.85)	0.088
		5 times/wk	3	5.51	0.063	63.73	0.45 (0.07–0.83)	0.020
	Duration	8 wk	5	8.12	0.087	50.72	0.46 (0.14–0.77)	0.004
		12 wk	2	5.31	0.021	81.15	0.33 (-0.41 to 1.07)	0.379

**Figure 4.**
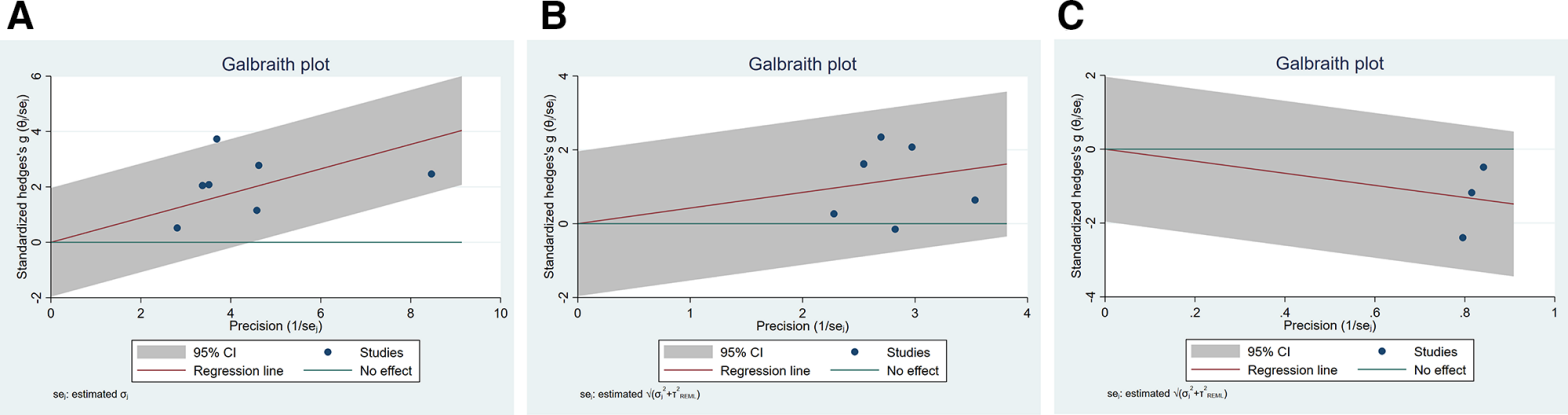
Galbraith plots (A: inhibition; B: working memory; C: cognitive flexibility). CI = confidence interval.

Galbraith plots were applied to visualize outliers with heterogeneity in the Meta-analysis. The X-axis represents individual ES standardized according to SE using the formula *y* = ES/SE, while the Y-axis displays precision (*x* = 1/SE). The regression diagonal line originates from the coordinate system’s origin (*x* = 0, *y* = 0). The approximate 95% CIs are delineated by 2 parallel lines at ± 2 units around the diagonal. As shown in Figure 4A, all points representing the included studies fall within the 95% CI apart from Yuping Zhu,^[44]^ indicating this study could be a source of heterogeneity.

To evaluate the robustness of the meta-analysis results, sensitivity analyses were performed. The pooled effect was analyzed by sequentially excluding individual studies. When the study by Yuping Zhu^[44]^ was excluded, the pooled ES of inhibition was Hedges’s *g* = 0.39, with a 95% CI of 0.24 to 0.55, *P* < .001. The *I*^2^ value decreased from 20.10% to 0.00%, indicating a significant reduction in heterogeneity and a statistically significant difference compared to the control group. The experimental site of Yuping Zhu^[44]^ was a community setting, suggesting it may have contributed to heterogeneity. Excluding this study resulted in a more stable pooled effect for Hedges’s *g* and *I*^2^, indicating a stable outcome. This suggested that exercise may enhance inhibition in individuals with SUD compared to those in the control group.

#### 3.4.2. Meta-analysis of the effect of physical exercise intervention on working memory in individuals with SUD

**Figure 5.**
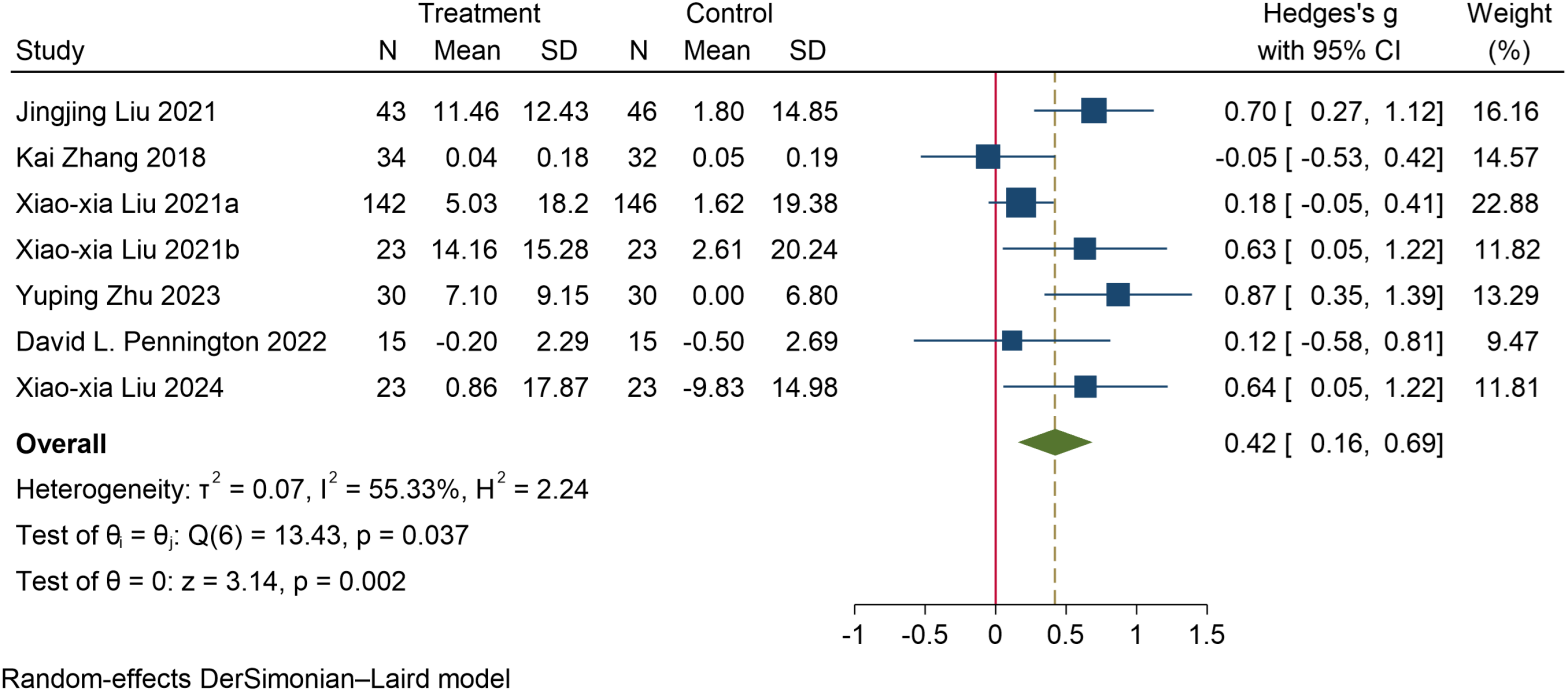
Forest plot of the effect of exercise on working memory. CI = confidence interval, SD = standard deviation.

Seven studies reported results on working memory.^[25,41–46]^ The heterogeneity analysis indicated moderate heterogeneity among the studies (*I*^2^ = 55.33%, *P* = .037). A random effects model analysis demonstrated that physical exercise significantly improved working memory in individuals with SUD (Hedges’s *g* = 0.42, 95% CI: 0.16 to 0.69, *P* = .002), showing a significant difference compared to the control group (Fig. 5). The 95% prediction interval for working memory ranged from −0.35 to 1.19. The prediction interval included zero, indicating that the true effect of exercise on working memory may vary substantially across study contexts.

To explore possible reasons for the heterogeneity, further subgroup analysis of potential adjustment variables was performed. As shown in Table 3, with single exercise time of 46 to 60 minutes, 5 times per week, or exercise duration of 8 weeks, physical exercise may improve the working memory of individuals with SUD (these subgroup findings are exploratory and should be interpreted as hypothesis-generating).

As shown in Figure 4B, all points representing the included studies fall within the 95% CI, indicating no significant heterogeneity among the studies included in the meta-analysis. A sensitivity analysis on the total intervention effect of working memory was conducted by sequentially excluding individual studies (Table 4). Excluding any single study resulted in a pooled effect Hedges’s g ranging from 0.35 to 0.50, an *I*^2^ range of 46.24% to 61.46%, and *P*-values all <.05. These results indicated low data sensitivity and no substantial change in the meta-analysis outcomes, suggesting stability and reliability.

**Table 4:** The pooled effect of subcomponents after excluding a single study.

Subcomponents	Study	Hedges’s *g*	95% CI	P	*I^2^* (%)
Inhibition	Dongshi Wang 2017^[40]^	0.47	0.27 to 0.67	< 0.001	27.49
	Jingjing Liu 2021^[25]^	0.46	0.25 to 0.67	< 0.001	26.32
	Ting Zhu 2021^[35]^	0.51	0.31 to 0.72	< 0.001	22.42
	Yuping Zhu 2023^[44]^	0.39	0.24 to 0.55	< 0.001	0.00
	Xiao-xia Liu 2021a^[42]^	0.55	0.35 to 0.75	< 0.001	18.92
	Xiao-xia Liu 2021b^[43]^	0.47	0.27 to 0.67	< 0.001	23.84
	David L. Pennington 2022^[45]^	0.50	0.30 to 0.69	< 0.001	25.08
	Xiao-xia Liu 2024^[46]^	0.47	0.27 to 0.67	< 0.001	23.84
Working memory	Jingjing Liu 2021^[25]^	0.37	0.08 to 0.65	0.011	52.32
	Kai Zhang 2018^[41]^	0.50	0.23 to 0.78	< 0.001	51.55
	Xiao-xia Liu 2021a^[42]^	0.49	0.19 to 0.80	0.001	47.29
	Xiao-xia Liu 2021b^[43]^	0.40	0.10 to 0.69	0.008	59.90
	Yuping Zhu 2023^[44]^	0.35	0.09 to 0.61	0.008	46.24
	David L. Pennington 2022^[45]^	0.46	0.17 to 0.75	0.002	61.46
	Xiao-xia Liu 2024^[46]^	0.40	0.10 to 0.69	0.008	59.80
Cognitive flexibility	Xiao-xia Liu 2021a^[42]^	0.25	-0.81 to 1.31	0.645	81.50
	David L. Pennington 2022^[45]^	0.90	0.68 to 1.12	< 0.001	0.00
	Xiao-xia Liu 2024^[46]^	0.35	-0.85 to 1.56	0.567	90.57

#### 3.4.3. Meta-analysis of the effect of physical exercise intervention on cognitive flexibility in individuals with SUD

**Figure 6.**
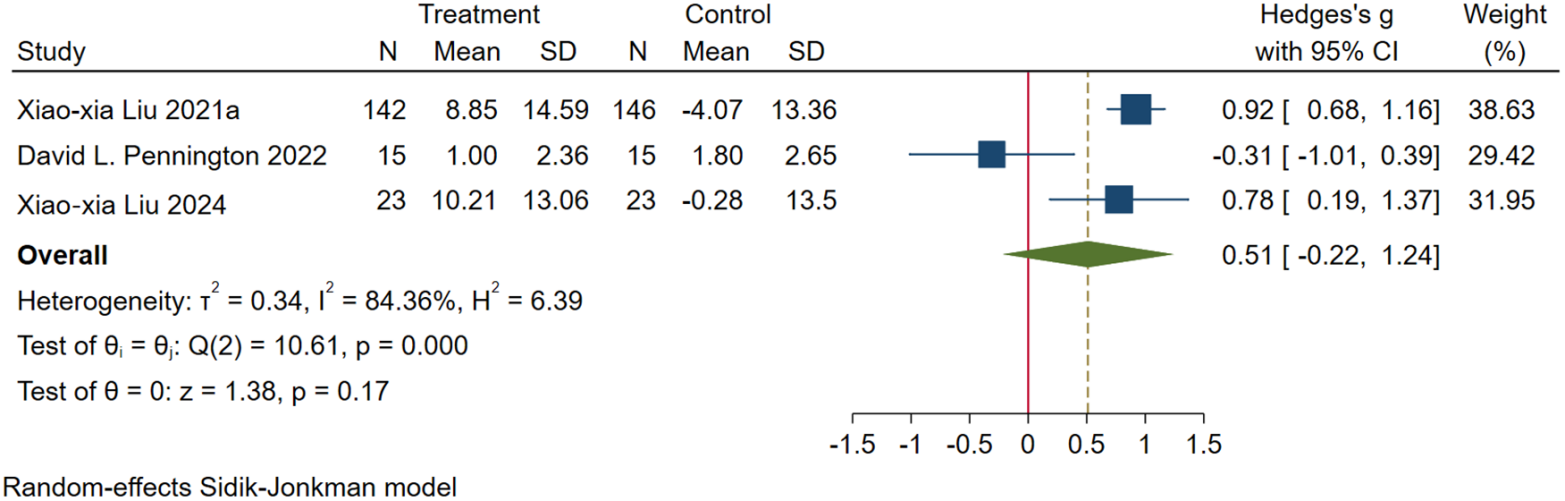
Forest plot of the effect of exercise on cognitive flexibility. CI = confidence interval, SD = standard deviation.

Three studies examined the impact of exercise on the cognitive flexibility subcomponent of executive function.^[42,45,46]^ Heterogeneity analysis revealed substantial heterogeneity among the studies (*I*^2^ = 84.36%, *P* = .000). Using a random-effects model, the meta-analysis indicated that the pooled ES was Hedges’s *g* = 0.51 (95% CI: −0.22 to 1.24, *P* = .170), showing no significant difference between the intervention and control groups (Fig. 6). A 95% prediction interval was not calculated for cognitive flexibility due to the small number of included studies (*k* = 3).

As the inclusion of only 3 studies, subgroup analyses could not be conducted. Subgroup analysis requires sufficient statistical power to detect differences. If there are only a few studies, statistical validity may be insufficient, leading to failure to draw meaningful conclusions.^[47]^ The ESs reported in the 2 studies (X. Liu & Wang, 2021; X.-X. Liu et al., 2024)^[42,46]^ were significant, while the study by David et al.^[45]^ produced an ES that was not significant.

As shown in Figure 4C, all points representing the included studies fall within the 95% CI, indicating no significant heterogeneity among the studies included in the meta-analysis.

To evaluate the robustness of the meta-analysis results, sensitivity analyses were conducted. The pooled effect was analyzed by sequentially excluding individual studies. After excluding David L. Pennington 2022,^[45]^ the pooled ES of cognitive flexibility was Hedges’s *g* = 0.90, with a 95% CI of 0.68 to 1.12, *P* < .001. The *I*^2^ decreased from 84.36% to 0.00%, indicating a significant reduction in heterogeneity and a statistically significant difference compared to the control group. The pooled ESs for Hedges’s g after excluding other individual studies ranged from 0.25 to 0.35, and the *I*^2^ ranged from 81.50% to 90.57%, *P* > .05 (Table 4). David L. Pennington 2022^[45]^ was the only study that included subjects with alcohol use disorder, suggesting that the type of substance may be a source of heterogeneity. Excluding this study resulted in more stable pooled effects for Hedges’s *g* and *I*^2^, indicating a stable outcome. This may suggest that the type of SUD contributed to heterogeneity. However, no firm conclusion can be drawn regarding methamphetamine use disorder due to the limited number of studies.

### 3.5. Publication bias

Given the limited literature in this study, indicators reported in fewer than 10 RCTs were not subjected to funnel plot analysis.^[48]^

### 3.6. Evaluation of the quality of evidence

**Figure 7.**
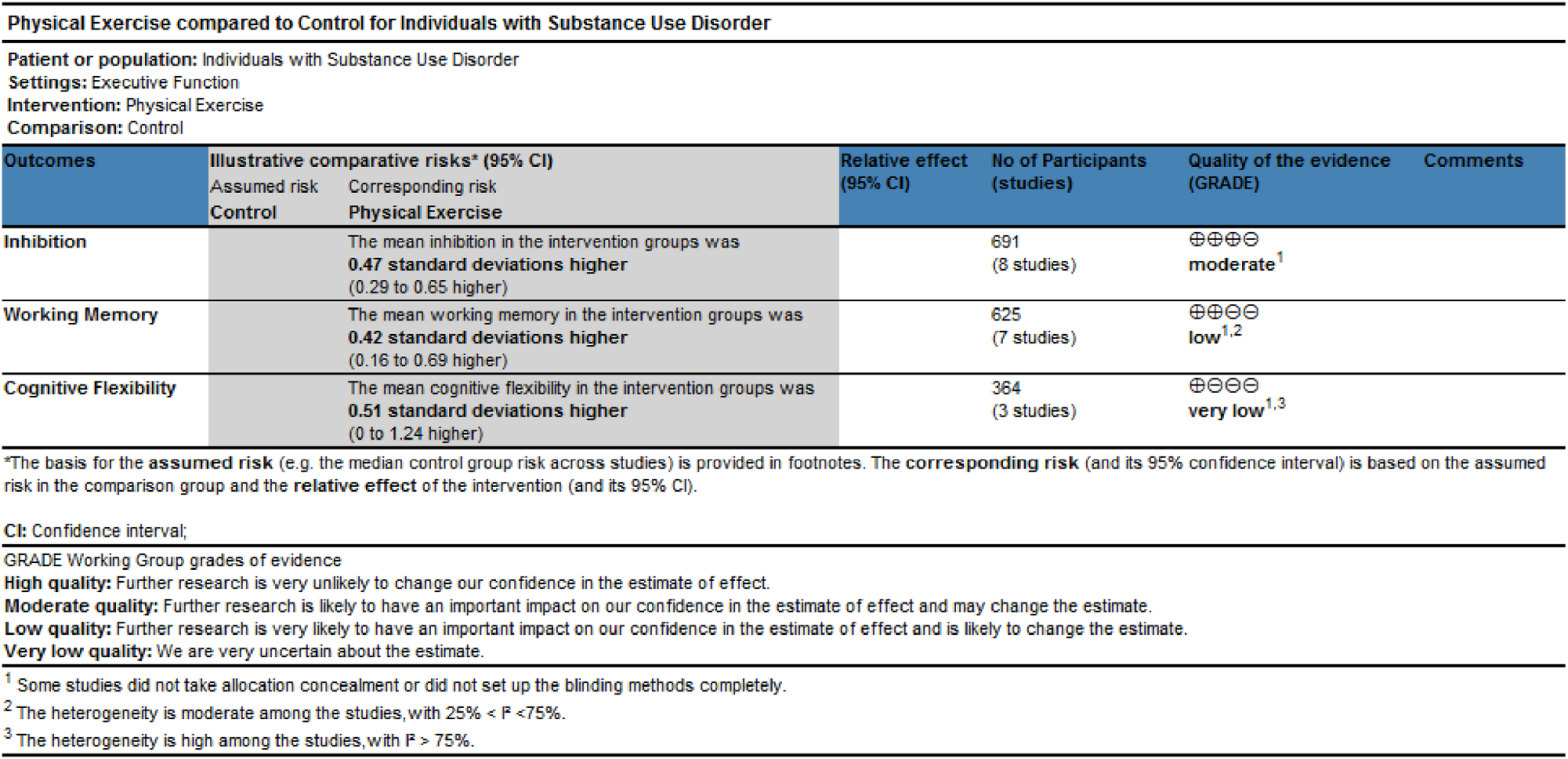
Evaluation of the quality of evidence. CI = confidence interval.

According to the Grading of recommendations assessment, development and evaluation (GRADE) Working Group methodology, the quality of evidence for each outcome was assessed across 5 domains: risk of bias, inconsistency, indirectness, imprecision, and other considerations. All outcomes began at high quality (all included studies were randomized controlled trials) and were rated as follows. Inhibition was rated as moderate, downgraded one level for serious risk of bias, as some studies lacked allocation concealment and adequate blinding of participants and outcome assessors (Fig. 2). Working memory was rated as low, downgraded 2 levels: one for serious inconsistency (*I*^2^ = 55.33%) and one for serious risk of bias from inadequate blinding (Fig. 2). Cognitive flexibility was rated as very low, downgraded 3 levels: one for serious inconsistency (*I*^2^ = 84.36%), and one for serious risk of bias from inadequate blinding (Fig. 2). The downgrading decisions with respect to risk of bias are consistent with the Cochrane Risk of Bias assessments summarized in Figure 2, which showed that the majority of included studies had unclear or high-risk of bias in the allocation concealment and blinding domains (see Figure 7).

## 4. Discussion

The findings demonstrate that physical exercise may improve executive function in individuals with SUD, with varying effects across the core components of inhibition, cognitive flexibility, and working memory. Physical exercise shows favorable improvements in inhibition and working memory, though these findings should be interpreted with caution given the heterogeneity and limited study numbers. However, it remains uncertain whether physical exercise can improve cognitive flexibility. The assessment of the quality of evidence for physical exercise in individuals with SUD indicated a moderate level of evidence for inhibition, a low level for working memory, and a very low level for cognitive flexibility.

The research findings revealed that physical exercise may enhance inhibition and working memory, aligning with previous findings.^[49,50]^ Inhibition reflects an advanced cognitive process that adaptively inhibits habitual or dominant behaviors in response to changing contingencies.^[51]^ The reward circuit and impulse model of inhibition are critical in drug addiction.^[52]^ Alterations in the mesolimbic dopaminergic reward system underlie the formation and consolidation of addictive behaviors.^[53]^ Long-term aerobic exercise could regulate neuroplasticity in the mesolimbic reward system.^[54]^ Drug use causes functional and structural anomalies in brain regions associated with inhibition, including the orbitofrontal region, anterior cingulate cortex, and insula.^[55]^ Moderate- and high-intensity aerobic exercise significantly increase activity in the prefrontal functional region.^[56]^ Aerobic exercise not only strengthens intercortical connections between the prefrontal and midfrontal regions at rest but also establishes a more complex neural network system, thereby enhancing inhibition.^[57]^ Substance use (e.g., methamphetamine, marijuana, etc) can lead to impaired working memory function. Short-term aerobic exercise also promotes neurogenesis, especially in the hippocampus, which is primarily associated with learning and memory. It increases the number of new neurons in the hippocampus, improving learning and memory functions.^[58]^ Additionally, moderate intensity aerobic exercise increases the production of neurotrophic factors, mitigates neuronal apoptosis in the hippocampus, and promotes more complex dendritic structures and longer dendritic lengths, which increase hippocampal volume and improve memory function.^[59]^ Aerobic exercise also facilitates the exit of neural stem cells from the quiescent phase, the proliferation of NSC and progenitor cells, the survival of new cells, the morphological development of immature neurons, and the integration of new neurons into the hippocampal circuit.^[60]^ Overall, these findings suggest aerobic exercise improves executive function and working memory in individuals with SUD by promoting neurogenesis, increasing neurotrophic factor production, and enhancing cortical neural network connectivity.

To further explore the dosage relationship of exercise on executive function, this study examined the regulatory effects of exercise on executive function in terms of single exercise time, exercise frequency, and exercise duration. Subgroup analyses showed that subgroups with different single exercise times and frequencies showed improvements in inhibition, though these findings should be interpreted as exploratory and hypothesis-generating. However, physical exercise could not improve inhibition in individuals with SUD when exercise duration was 4 weeks. Few studies have concluded that exercise for 4 weeks or less improves inhibition. It is hypothesized that this is because the exercise duration is too short to produce a significant cumulative effect. On the other hand, physical exercise was found to enhance the working memory of individuals with SUD when the single exercise time was 46 to 60 minutes, or the exercise frequency was 5 times per week, or the exercise duration was 8 weeks. Similar results in some studies^[61–63]^ were observed that physical exercise in these exercise conditions was effective in improving working memory. Notably, though subgroup analyses suggested a potential “dose-response” relationship regarding the effects of physical activity interventions on inhibition and working memory in individuals with SUD, these findings remain exploratory and hypothesis-generating. Consistent with standard GRADE methodology, the evidence level was not upgraded on the basis of these subgroup findings. They do not yet establish a definitive optimal dose, as the current subgroup analyses were limited by small sample sizes.

Although the results of this study showed no significant difference between the treatment and control groups in terms of improving cognitive flexibility. This result contrasts with previous findings.^[64]^ However, it still does not indicate that physical exercise fails to improve cognitive flexibility in individuals with SUD. Given the small number of included studies (*k* = 3), the high heterogeneity (*I*^2^ = 84.36%), and the resulting limited statistical power, these findings should be interpreted with caution and considered as hypothesis-generating rather than definitive. Therefore, more high quality RCTs are warranted for validation in the future.

Results of sensitivity analyses indicated that addictive substances may contribute to heterogeneity. Sensitivity analyses suggested that the study by David L. Pennington et al.^[45]^ May be the source of heterogeneity. This study uniquely included individuals with alcohol addiction. Alcohol addiction may damage the brain, and although physical exercise can reverse long-term hippocampal damage by enhancing natural self-repair processes,^[65]^ abstinence alone leads to significant self-recovery. A previous study suggests that binge drinking decreases neurogenesis in the dentate gyrus, while the hippocampus increases cytogenesis during abstinence.^[66]^ A recent study indicated that executive function can self-restore in individuals with alcohol use disorder after 6 to 12 months of abstinence.^[67]^ In addition, in the sensitivity analyses of inhibition, the study by Yuping Zhu et al.^[44]^ was found to be a possible source of heterogeneity. Methodological heterogeneity was evident as the intervention site for this study was a community-based, non-compulsory drug treatment program, unlike other studies conducted in compulsory drug treatment centers and specialized rehabilitation centers. The intervention site’s nature may introduce heterogeneity, as the effects of physical exercise on inhibition in an unenclosed and supervised environment could be confounded by external factors. In a non-compulsory setting, many uncontrollable factors and potential interferences exist. Drug users, without considering catastrophic consequences, especially when exposed to salient drug-related cues, may lack inhibition due to unfettered motivation.^[68]^ For instance, exposure to drug-related images or videos can stimulate intrinsic motivation to take drugs, reducing inhibition abilities.^[69]^

Nonetheless, this study has several limitations: The extracted data lacks information on several variables, such as gender, duration of using substance, and types of addictive substances just being methamphetamine and alcohol, which limits the generalizability of the results. Thus, future investigations including these issues are needed. The small number of studies included in this review limits the analysis of possible moderators that could impact the results obtained. Some study results exhibit significant heterogeneity, which potentially affects the reliability of the conclusions and necessitates cautious interpretation. and Due to limited data on cognitive flexibility, the results in this domain should also be interpreted with caution.

## 5. Conclusion

Physical exercise may effectively enhance inhibition and working memory in individuals with SUD, though conclusions should be interpreted cautiously given study limitations. However, due to the small number of studies, the current findings on cognitive flexibility are still inconclusive, and more randomized trials with high quality and large samples are needed in the future to further clarify it.

## Author contributions

**Data curation:** Jiawei Chen, Xiaofei Zhang, Siqin Zeng.

**Methodology:** Jiawei Chen, AV Kabachkova.

**Software:** Xiaofei Zhang.

**Formal analysis:** Siqin Zeng.

**Supervision:** Wenwu Xiao.

**Writing – original draft:** Jiawei Chen.

**Writing – review & editing:** AV Kabachkova, Wenwu Xiao.


