# Supplementary material for: Effect of physical exercise on executive function in individuals with substance use disorder: A systematic review and meta-analysis
Source: Medicine (Baltimore). 2026 Jul 17;105(29):e49771. doi: 10.1097/MD.0000000000049771 (PMC13384601; doi:10.1097/MD.0000000000049771)
Supplement: Supplementary file 1 [file medi-105-e49771-s001.docx]

****Web of Science (WOS)****

#1 TS=(substance use disorder)

#2 (((AB=(substance abuse)) OR AB=(drug abuse)) OR AB=(drug addiction)) OR AB=(drug use disorder)

#3 #1 OR #2

#4 TS=(exercise)

#5 ((((((AB=(physical exercise)) OR AB=(physical activity)) OR AB=(training)

#6 #4 OR #5

#7 TS=(cognition)

#8 (((((AB=(executive function)) OR AB=(cognitive function)) OR AB=(inhibition)) OR AB=(inhibitory control)) OR AB=(switching function)) OR AB=(working memory)) OR AB=(cognitive flexibility)

#9 #7 OR #8

#10 TS=(randomized controlled trial)

#11 ((AB=(randomized)) OR AB=(controlled)) OR AB=(trial)

#12 #10 OR #11

#13 #3 AND #6 AND #9 AND #12

****Pubmed****

**((("Substance-Related Disorders"[Mesh]) OR (substance abuse[Title/Abstract] OR drug abuse[Title/Abstract] OR drug addiction[Title/Abstract] OR drug use disorder[Title/Abstract] OR substance use disorder[Title/Abstract]))**

**AND**

**(("Cognition"[Mesh]) OR (executive function[Title/Abstract] OR cognitive flexibility[Title/Abstract] OR cognitive function[Title/Abstract] OR inhibitory control[Title/Abstract] OR inhibition[Title/Abstract] OR switching function[Title/Abstract] OR working memory[Title/Abstract]))**

**AND**

**(("Exercise"[Mesh]) OR (aerobic exercise[Title/Abstract] OR cycling[Title/Abstract] OR walking[Title/Abstract] OR physical activity[Title/Abstract] OR training[Title/Abstract]))**

**AND**

**("Randomized Controlled Trial"[Publication Type] OR randomized[Title/Abstract] OR randomised[Title/Abstract] OR**

**controlled[Title/Abstract] OR trial[Title/Abstract]))**

****Cochrane library****

**#1 MeSH descriptor: [Substance-Related Disorders] explode all trees**

**#2 ("substance abuse" OR "drug abuse" OR "drug addiction" OR "drug use disorder" OR "substance use disorder"):ti,ab,kw**

**#3 #1 OR #2**

**#4 MeSH descriptor: [Exercise] explode all trees**

**#5 ("physical exercise" OR "aerobic exercise" OR "cycling" OR "walking" OR "physical activity" OR "training"):ti,ab,kw**

**#6 #4 OR #5**

**#7 MeSH descriptor: [Cognition] explode all trees**

**#8 ("executive function" OR "cognitive flexibility" OR "cognitive function" OR "inhibitory control" OR "inhibition" OR "switching function" OR "working memory"):ti,ab,kw**

**#9 #7 OR #8**

**#10 MeSH descriptor: [Randomized Controlled Trial] explode all trees**

**#11 ("randomized" OR "randomised" OR "controlled" OR "trial"):ti,ab,kw**

**#12 #10 OR #11**

**#13 #3 AND #6 AND #9 AND #12**

**Embase**

('drug dependence'/exp OR 'substance abuse':ab,ti OR 'substance use disorder':ab,ti OR 'drug abuse':ab,ti OR 'drug addiction':ab,ti OR 'drug use disorder':ab,ti) AND ('cognition'/exp OR 'executive function':ab,ti OR 'cognitive flexibility':ab,ti OR 'cognitive function':ab,ti OR 'inhibitory control':ab,ti OR 'switching function':ab,ti OR 'working memory':ab,ti) AND ('randomized controlled trial'/exp OR 'randomized controlled trial' OR randomized:ab,ti OR randomised:ab,ti OR controlled:ab,ti OR trial:ab,ti) AND ('exercise'/exp OR 'aerobic exercise':ab,ti OR 'physical exercise':ab,ti OR 'cycling':ab,ti OR 'walking':ab,ti OR 'physical activity':ab,ti OR 'training':ab,ti)
